# A Phase I Trial of VEGF-A Inhibition Combined with PD-L1 Blockade for Recurrent Glioblastoma

**DOI:** 10.1158/2767-9764.CRC-22-0420

**Published:** 2023-01-25

**Authors:** Daniel Chiu, Jingjing Qi, Tin Htwe Thin, Monica Garcia-Barros, Brian Lee, Mary Hahn, John Mandeli, Puneet Belani, Kambiz Nael, Omid Rashidipour, Saadi Ghatan, Constantinos G. Hadjipanayis, Raymund L. Yong, Isabelle M. Germano, Rachel Brody, Nadejda M. Tsankova, Sacha Gnjatic, Seunghee Kim-Schulze, Adília Hormigo

**Affiliations:** 1 Icahn School of Medicine at Mount Sinai, New York, New York.; 2 Departments of Oncological Sciences, Medicine (Hematology/Oncology), and Pathology and Precision Immunology Institute, Human Immune Monitoring Center, Tisch Cancer Institute, Icahn School of Medicine at Mount Sinai, New York, New York.; 3 Department of Pathology, Mount Sinai Medical Center, New York, New York.; 4 Environmental Medicine and Public Health, Mount Sinai Medical Center, New York, New York.; 5 Diagnostic, Molecular & Interventional Radiology, Mount Sinai Medical Center, New York, New York.; 6 Department of Radiological Sciences, David Geffen School of Medicine, University of California Los Angeles, Los Angeles, California.; 7 Departments of Neurological Surgery and Oncological Sciences, Icahn School of Medicine at Mount Sinai, New York, New York.; 8 Montefiore Einstein Cancer Center, and Departments of Hematology-Oncology, Neurosurgery, Microbiology & Immunology, Albert Einstein College of Medicine, Bronx, New York.

## Abstract

**Purpose:**

The treatment of glioblastoma (GBM) poses challenges. The use of immune checkpoint inhibition (ICI) has been disappointing as GBM is characterized by low mutational burden and low T-cell infiltration. The combination of ICI with other treatment modalities may improve efficacy.

**Patient and Methods:**

Patients with recurrent GBM were treated with avelumab, a human IgG1 antibody directed against PD-L1 (part A), or avelumab within a week after laser interstitial thermal therapy (LITT) and continuation of avelumab (part B). Bevacizumab was allowed to be combined with ICI to spare steroid use. The primary objective was to characterize the tolerability and safety of the regimens. The secondary objectives included overall survival, progression-free survival (PFS), signatures of plasma analytes, and immune cells.

**Results:**

A total of 12 patients (median age 64; range, 37–73) enrolled, five in part A and seven in part B. Two serious adverse events occurred in the same patient, LITT treated, not leading to death. The median survival from enrollment was 13 months [95% confidence interval (CI), 4–16 months] with no differences for part A or B. The median PFS was 3 months (95% CI, 1.5–4.5 months). The decrease in MICA/MICB, γδT cells, and CD4^+^ T cell EMRA correlated with prolonged survival.

**Conclusions:**

Avelumab was generally well tolerated. Adding bevacizumab to ICI may be beneficial by lowering cytokine and immune cell expression. The development of this combinatorial treatment warrants further investigation. Exploring the modulation of adaptive and innate immune cells and plasma analytes as biomarker signatures may instruct future studies in this dismal refractory disease.

**Significance:**

Our phase I of PD-L1 inhibition combined with LITT and using bevacizumab to spare steroids had a good safety profile for recurrent GBM. Developing combinatory treatment may help outcomes. In addition, we found significant immune modulation of cytokines and immune cells by bevacizumab, which may enhance the effect of ICI.

## Introduction

Glioblastoma (GBM), the most frequent malignant brain tumor in adults, is a devastating disease with a median survival of 14.6 months for patients treated with standard radiotherapy and chemotherapy and reaching a median of 21.7 months when selecting patients whose tumors have epigenetic silencing of the O6-methylguanine-DNA methyltransferase (MGMT) DNA repair gene by promoter methylation (1).

The disease invariably recurs. There is no first-line treatment for recurrent disease, and patients are left with the option of a clinical trial if available or the physician's best choice for a particular patient. Studies utilizing lomustine chemotherapy combined with bevacizumab versus lomustine alone at first recurrence reported an improvement of progression-free survival (PFS) of 4.2 versus 1.5 months, but no survival advantage for the combination with a median overall survival (OS) of 9.1 versus 8.6 months for lomustine alone (2). The treatment of recurrent GBM continues to be an unmet medical need.

The use of immune checkpoint inhibition (ICI), which removes T-cell activation inhibitory signals, unleashing T cells to eradicate tumor cells, has been groundbreaking (3). Several ICI have been approved with durable responses and even cures for the responders. However, those responders represent a small number for each disease (3). Furthermore, some types of cancers, including GBM, have been remarkably resistant to treatment with ICI. A phase III clinical trial in which patients with recurrent GBM were randomized to nivolumab or bevacizumab showed a similar median survival of 9.5 and 10 months, respectively (4). In truth, the relatively low mutation burden (5), low levels of PD-L1 expression (6), immunosuppressive tumor microenvironment (TME) with abundant myeloid-derived suppressor cells (MDSC; ref. 7), T-cell dysfunction with exhaustion (8), and sequestration of T cells in the bone marrow (9), render GBM refractory to ICI.

Nevertheless, intratumor and immune landscape heterogeneity may benefit the treatment of GBM with ICI (10). One study using neoadjuvant pembrolizumab to treat recurrent GBM (11) or expression of PD-L1 by the tumor (12) improved the median survival to 13.7 and 13.1 months, respectively, compared with historical controls using lomustine combined with bevacizumab (2). The treatment efficacy of ICI can be broadened by combining immunotherapy treatments (3) or combining ICI with other modalities. In addition, in tumors such as GBM with low neoantigen load, a strategy that increases tumor cell death should increase the exposure of neoantigens to antigen-presenting cells that cross-present to tumor-reactive T cells. On the basis of this assumption, we utilized laser interstitial thermal therapy (LITT), a minimally invasive procedure that can accurately ablate tumor tissue in real time by MRI guidance and promote cell death by heating the tumor to high temperatures to increase the neoantigen load. While the first group of patients was treated with avelumab, an IgG1 mAb that binds PD-L1 (part A), the second was treated with LITT, followed by avelumab.

## Materials and Methods

### Patients

Eligible patients were 18 years of age or older with a first recurrence of wild-type GBM (Supplementary Table S1), Karnofsky score ≥60, and had a lesion(s) deemed adequate to undergo the LITT procedure. A maximum of two lesions with a sectional diameter of at least >1 cm but ≤3 cm in a trajectory felt safe by the neurosurgeon performing the procedure were selected, avoiding transgressing a ventricle and eloquent structures. A stable daily dexamethasone dose of ≤4 mg was required for enrollment to avoid higher doses of steroids that could abrogate the effect of the immunotherapy. The exclusion criteria were prior implantation of intracavitary carmustine wafers, significant active autoimmune disease, infection, immunodeficiency, concurrent cancer, or prior ICI. The study was conducted in accordance with the Declaration of Helsinki and the International Council for Harmonization Guidelines for Good Clinical Practice. The Institutional Review Board of the Icahn School of Medicine at Mount Sinai, where the study was conducted, approved the study protocol. All participants provided written informed consent prior to enrolment.

### Trial Design and Treatment

This study was a prospective open-label, nonrandomized phase I study exploring the safety and tolerability of avelumab, an IgG1 mAb that, by binding PD-L1, prevents binding to the PD-1 receptor. The trial was registered on clinicaltrials.gov (NCT03341806). The authors performed the study at a single institution. The study enrolled patients in two successive safety phases: the first cohort of avelumab alone (part A) and the second cohort of MRI-guided LITT followed by avelumab (part B). The study followed a 3+3 design if no more than 0/3 or 1/6 patients experienced dose-limiting toxicities (DLT) in part A. A DLT was defined as a grade ≥3 adverse reaction suspected to be related to avelumab. Avelumab was administered initially at a dose of 10 mg/kg intravenous every 2 weeks and subsequently changed to a 60-minute infusion of 800 mg after the FDA authorized a flat dose for approved indications. Avelumab was given within a week after LITT and every 2 weeks after that until disease progression, intolerable toxicity, or withdrawal of patient consent. However, patients could continue the study treatment following evidence of disease progression if deemed appropriate at the investigator's discretion. If patients became symptomatic and required either initiation or increased doses of dexamethasone, they were allowed to remain in the study and treated with bevacizumab to avoid the immunosuppressive effect of steroid use.

### Endpoints

The primary endpoints were the safety and tolerability of avelumab after LITT and the objective response rate to treatment. The secondary endpoints included OS, defined as the time from enrollment to death from any cause, and PFS, defined as the time from enrollment to documented disease progression. Other secondary endpoints included the detection of protein biomarkers in serum samples, changes in circulating immune cells, and pathologic and immunologic analyses of the tumor specimens.

### Study Assessments

Feasibility, side effects, and adverse events (AE) were consistently monitored. All subjects who received at least one dose of avelumab were analyzed for safety. AEs were assessed according to the NCI Common Terminology Criteria for Adverse Events version 4.03. In addition, two independent neuroradiologists evaluated the response rate by MRI using the modified radiophraphic response assessment in neuro-oncology (mRANO) criteria every 8 weeks. The proportion of patients who achieved complete response or partial response (PR) determined the objective response rate. OS and median PFS were determined using the Kaplan–Meier method and calculated from enrollment to death from any cause or progression on MRI. Estimating median survival time and median PFS, 95% confidence intervals (CI) were calculated and included sex as a variable. Patients who did not progress or die were censored on the date of the last assessment.

### Analysis Plan for Secondary Endpoints

An Olink multiplex proximity extension assay platform with an immuno-oncology panel (Olink Bioscience) was used according to the manufacturer's instructions. The panel includes 92 proteins associated with immune responses, inflammatory cytokines, chemokines, and soluble immune checkpoint molecules. Peripheral blood mononuclear cells were analyzed by cytometry using time-of-flight (CyTOF) with reference sample spike-in and palladium-based mass tag cell barcoding of individual samples. The Astrolabe platform was used to annotate and analyze specific cell populations. Plasma cytokines, chemokines, and circulating immune cells were assessed at three discrete timepoints (pretreatment baseline and two timepoints after avelumab treatment). Potential biomarkers associated with the activity of avelumab alone and LITT followed by avelumab with or without bevacizumab were investigated separately and correlated with survival. Normalized protein expression (NPX) and phenotypic frequency were used to identify changes in individual proteins across the sample set.

Formalin-fixed paraffin-embedded 3-μm sections from archival tissue at initial diagnosis and when available from LITT procedure or a subsequent re-resection were used for single, dual, or multi-chromogen sequential IHC. IHC was performed using the Ventana Discovery Ultra (Roche Diagnostics). This system allows automated baking, deparaffinization, and cell conditioning. Single staining was performed using the RUO Discovery Multimer V2 (v0. 00.0083) and dual and multiplex chromogens using RUO Discovery Universal (v21.00.0019). Prediluted primary antibodies were obtained from Roche Diagnostics (Supplementary Table S2): Ki-67 (30–9) (790–4286) (RRID:AB_2631262); CD3 (2GV6) (790–4341) (RRID:AB_2335978); CD163 (MRQ-26) (760–4437) (RRID:AB_2335969); CD45 (LCA) (2B11 and PD7/26) (760- 4279) (RRID:AB_2927457); CD68 (KP-1) (790–2931) (RRID: AB_2335972); CD8 (SP57) (790–4460) (RRID: AB_2335985); and CD31 (JC70) (760–4378) (RRID:AB_2927455). PD-L1 (28–8) (ab205921) (RRID: AB_2687878) from Abcam was used (dilution, 1:50). All primary antibodies were incubated for 60 minutes at 37°C. OmniMap HRP or NP DISCOVERY (RUO; Roche Diagnostics) was used as the secondary antibody. The signal was detected using Discovery OmniMap (DAB, purple, teal, or yellow). Mayer hematoxylin was used for nuclear counterstaining. Whole tissue sections on the slide were converted into high-resolution digital data using a NanoZoomer S210 Digital slide scanner (Hamamatsu).

The HALO image analysis platform was used for quantitative tissue analysis (Indica Labs, Inc.), using the Multiplex IHC module and color deconvolution to separate chromogenic stains to prepare for quantitative analysis.

### Correlative Studies Statistical Analysis

R software (version 4.0.3) was used for nonparametric statistical analysis. The Mann–Whitney *U* test was performed to compare two groups, and the Kruskal–Wallis test was used to compare three or more groups. The Kaplan–Meier curve was used to plot the survival and PFS curves. The log-rank test was performed to evaluate differences between subgroups. The Benjamini–Hochberg method was used to correct for multiple comparisons.

### Data Availability

The clinical data generated in this study are not publicly available due to patient privacy requirements. Other data generated in this study are available within the article and its Supplementary Data.

## Results

### Characteristics of the Patients

From June 2018 to November 2019, we enrolled 12 patients with isocitrate dehydrogenase–wildtype glioblastoma in the study; 5 were analyzed in part A and 7 in part B. The first patient in part A withdrew from the intervention of receiving avelumab after experiencing rigors during the first infusion, a known side effect of the drug that was easily controlled with supportive measures. He was replaced by another patient. He continued treatment with nivolumab at his request, another drug that blocks the PD-1-PD-L1 pathway. After enrolling the third patient, 2 other patients that were eligible for the trial returned to the clinic simultaneously to sign consent to participate in part A of the study. It was felt that denying participation to one of them would be unethical. Part B followed part A. As 1 of 3 experimented a DLT, 3 other patients were enrolled. As only 1 of 6 experienced a DLT in part B, we initiated the expansion cohort and treated the first patient. Unfortunately, with the COVID-19 pandemic, elective surgical procedures were halted in 2020 in New York City. Therefore, we were unable to continue patient accrual, and we were forced to close the study remaining open for follow-up of the enrolled patients and completing the correlative studies. In total, there were 7 men (58%) and 5 women (42%), with a median age of 64 years (range, 37–73 years; Table 1).

**TABLE 1:** Patient demographics and baseline characteristics

Characteristic	Group A: Avelumab (*N* = 5)	Group B: LITT + Avelumab (*N* = 7)	Total (*N* = 12)
Gender, no. (%)			
Male	4 (80)	3 (43)	7 (58)
Female	1 (20)	4 (57)	5 (42)
Median age, years (range)	51 (37–78)	66 (58–73)	64 (37–78)
Race, no. (%)			
White	5 (100)	2 (29)	7 (58)
Black	0 (0)	2 (29)	2 (17)
Hispanic	0 (0)	1 (14)	1 (8)
Asian	0 (0)	2 (29)	2 (17)
Karnofsky performance score (KPS)			
<70	0 (0)	2 (29)	2 (17)
≥70	5 (100)	5 (71)	10 (83)
Years from initial diagnosis to recurrence, median (range)	1.0 (0.3–6.3)	0.8 (0.6–1.1)	0.9 (0.3–6.3)
Days from LITT to avelumab initiation, median (range)	N/A	5 (2–6)	5 (2–6)
Avelumab infusion			
No. of patients (%)	5 (100)	7 (100)	12 (100)
Median no. of cycles (range)	4 (1–16)	8 (1–58)	7.5 (1–41)
Nivolumab infusion			
No. of patients (%)	2 (40)	1 (14)	3 (25)
Median no. of cycles (range)	22 (21–23)	10	21 (10–23)
Corticosteroid use, no. (%)	3 (60)	5 (71)	8 (66)
Post-LITT, Pre-avelumab	0 (0)	2 (40)	2 (25)
During avelumab treatment	2 (67)	1 (20)	3 (38)
After discontinuation of avelumab	1 (33)	2 (40)	3 (38)
Corticosteroid dose, no. (%)			
<4 mg/day	1 (33)	0 (0)	1 (13)
≥4 mg/day	2 (67)	5 (100)	7 (88)
Bevacizumab infusion, median no. of cycles (range)	5 (0–17)	12 (0–53)	10 (0–53)

**FIGURE 1:**
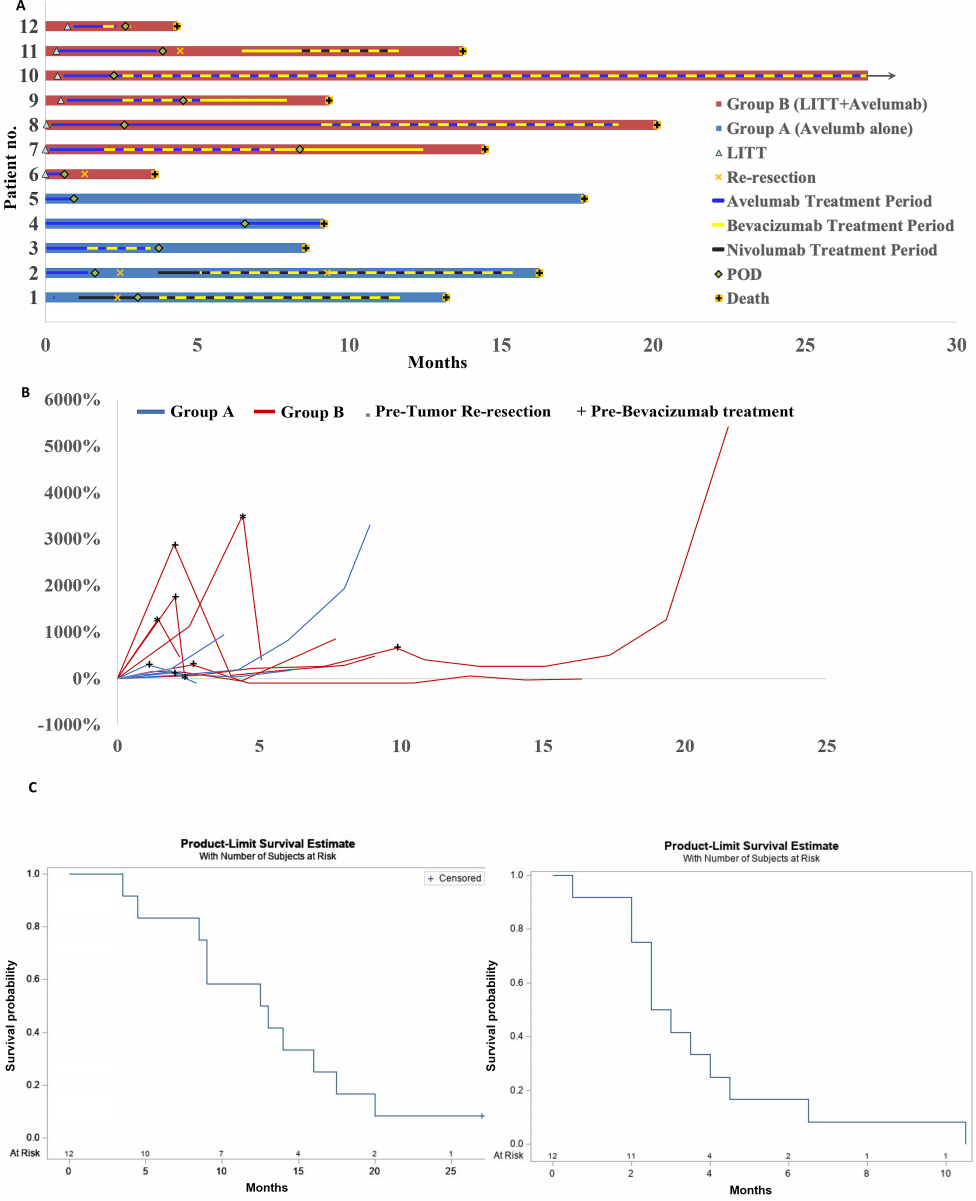
Disease course, response to treatment, and outcomes. **A,** Shows treatments received by patients with recurrent glioblastoma, the duration of the treatments to closing the study with the key within the plot showing all symbols and color coding, and each bar representing a patient. **B,** Shows the percent change of tumor growth or reduction from baseline at enrollment and through the treatment. **C,** Indicates survival (left) and PFS (right) Kaplan–Meier curves.

All patients were previously treated with standard radiotherapy and concurrent temozolomide, followed by adjuvant temozolomide prior to the first tumor recurrence. Patients in part A completed a median of four cycles of avelumab (range, 1–16) compared with a median of eight cycles (range, 3–58) in part B. Two patients in part A received neoadjuvant ICI (Fig. 1A); the one who withdrew from treatment after experiencing rigors with the first cycle of avelumab received two treatments of off-label nivolumab followed by re-resection of the tumor and resuming nivolumab for 21 infusions and 17 of bevacizumab; the other patient underwent a re-resection after four treatments of avelumab and had progression of the disease, and was subsequently treated with 23 treatments of nivolumab and 15 of bevacizumab. A patient in group B received only one dose of avelumab, underwent re-resection, and declined further treatment (Fig. 1A). Another patient in group B received eight infusions of neoadjuvant avelumab, underwent re-resection, which revealed tumor progression and received 10 infusions of nivolumab and eight of bevacizumab. Treatment with nivolumab was used off-labeled after progression of disease in those 3 patients. Nivolumab was considered a reasonable option that we had experience treating other patients with recurrent GBM.

Six patients received bevacizumab in addition to avelumab (Fig. 1A); 3 patients in part A received a median of five cycles of bevacizumab (range, 0–17), and 5 in part B received a median of 12 cycles (range, 0–53). Eight patients (67%) received dexamethasone during their treatment course (Table 1). Two in part B received 8 to 40 mg daily from days 1–3 after LITT. Another in part B and 2 patients in part A were treated with dexamethasone during their avelumab treatment, at dosages ranging from 0.5 to 8 mg daily. The remaining 3 patients received dexamethasone only after avelumab was discontinued.

### Safety and Feasibility

All patients received at least one dose of avelumab. There were no DLTs observed from using avelumab as a single agent, but we observed it in one patient with the combined treatment. The patients in part B were treated with avelumab between days 2 and 6 after LITT.

There were no unexpected AEs associated with avelumab in the patients enrolled in the trial, and no AEs led to death (Table 2). None of the patients had immune-related AEs, except one with a persistent dry mouth possibly related to avelumab. There were two episodes of severe AEs (SAE) occurring in the same patient (Table 2), consisting of right-sided weakness and fall and progressive aphasia, and thought to be related to the combination of LITT followed by avelumab due to increased vasogenic edema seen on MRI. The SAEs were grade 3 in severity and required hospitalization twice, each following an infusion, with a delay in administering subsequent avelumab treatment after the first SAE and discontinuation of treatment after his second SAE. Three patients experienced one, and 3 patients experienced two avelumab-related AEs. The most frequent AEs were rash in 3 patients and rigors in 2.

**TABLE 2:** Incidence of AEs and AEs suspected to be related to avelumab or LITT plus avelumab (A or LITT+A)

	No of patients (%)		
Adverse event	Any grade	Grade ≥3	A or LITT+A any grade
Clinical events			
Weakness	7 (58)	2 (17)	1 (8)
Nausea and/or vomiting	5 (42)	0 (0)	1 (8)
Headache	4 (67)	0 (0)	0 (0)
Fatigue	4 (67)	0 (0)	0 (0)
Dysphagia	3 (25)	0 (0)	0 (0)
Constipation	3 (25)	0 (0)	0 (0)
Gait disturbance	3 (25)	0 (0)	0 (0)
Rash	3 (25)	0 (0)	3 (25)
Rigors after infusion	3 (25)	0 (0)	2 (17)
Aphasia	2 (17)	1 (8)	1 (8)
Visual disturbance	2 (17)	0 (0)	0 (0)
Memory impairment	2 (17)	0 (0)	0 (0)
Pain	2 (17)	0 (0)	0 (0)
Anorexia	2 (17)	0 (0)	0 (0)
Ataxia	2 (17)	0 (0)	0 (0)
Dizziness	2 (17)	0 (0)	0 (0)
Fall	2 (17)	1 (8)	0 (0)
Paresthesias	2 (17)	0 (0)	0 (0)
Dysarthria	1 (8)	0 (0)	0 (0)
Cough	1 (8)	0 (0)	0 (0)
Cramp (leg)	1 (8)	0 (0)	0 (0)
Dry mouth	1 (8)	0 (0)	1 (8)
Hypersomnia	1 (8)	0 (0)	0 (0)
Hypertension	1 (8)	0 (0)	0 (0)
Psych disturbance	1 (8)	0 (0)	0 (0)
Shortness of breath	1 (8)	0 (0)	0 (0)
Somnolence	1 (8)	0 (0)	0 (0)
Laboratory events			
Lymphocyte decrease	3 (25)	0 (0)	0 (0)
Creatinine increase	3 (25)	0 (0)	0 (0)
Thrombocytopenia	1 (8)	0 (0)	0 (0)

### Treatment Response and Survival

There was no objective response to avelumab or LITT followed by avelumab treatment as determined by mRANO criteria (Fig. 1B). The median PFS was 2.75 months (95% CI, 2–4.5 months) for the 12 patients (Fig. 1C, right), 3 months (95% CI, 1.5–6.5 months) for patients in part A and 2.5 months (95% CI, 0.5–4.5 months) for patients in part B. The percentage of patients that attained a 6-month PFS was 16.5%. The median PFS was 3 months (95% CI, 1.5–3.5 months) for men and 2.5 (95% CI, 0.5–10.5 months) for women (Supplementary Fig S1).

Kaplan–Meier analysis revealed that the median OS for the 12 patients from the date of enrollment in the study was 12.75 months (95% CI, 4.5–17.5 months; Fig. 1C, left), 13 months in part A and 13.5 (95% CI, 3.5–19.5 months) for patients in part B. In addition, 6 patients (3 men and 3 women) survived longer than 12 months. Kaplan–Meier analysis showed that the median survival was 13 months for men and 14.5 months for women (Supplementary Fig S1). Three of these women had the most prolonged survival at the time of analysis (16, 19.5 months, with one alive at 27 months at the study's closing). They had a PR when bevacizumab was combined with avelumab, with an overall response rate of 25% only when bevacizumab was utilized.

### Tumor Characteristics

**FIGURE 2:**
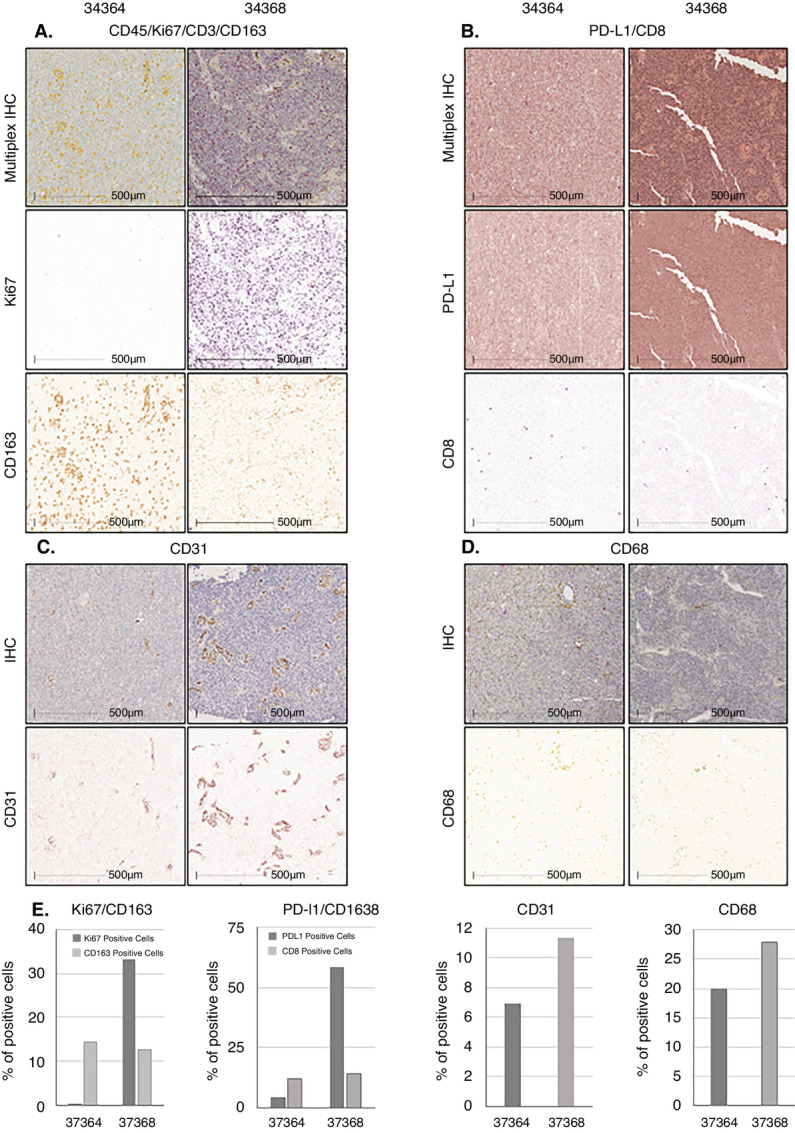
Chromogenic multiplex expression analysis of different biomarkers in formalin-fixed paraffin-embedded tissue of a patient's tumor with a survival of 13 months (34364) and another 3.5 months (34368) after treatment and quantitative analysis using Halo image analysis platform. **A,** Representative field for chromogenic multiplex for D45(brown)/Ki67(purple)/CD3(teal)/CD163(yellow) for case 34364 (left) and 34368 (right) tissues. Color deconvolution for each marker was obtained using HALO image analysis, and quantitative analysis for Ki67 and CD163 is shown in **E** (CD45 and CD3 color deconvolution and HALO quantification is shown in Supplementary Fig. S2). **B,** Representative field for chromogenic multiplex for PD- L1 (brown)/CD8 (purple) with their respective deconvolution images and quantification on **E**. **C,** Representative field for CD31 IHC in brown with image deconvolution with quantitative analysis in **E**. **D,** Representative field for CD68 IHC in yellow with image deconvolution with quantitative analysis in **E**.

At the initial resection, seven tumors had *EGFR* amplification, five were not amplified, seven had an unmethylated *MGMT* promoter, four were methylated, and one had an unknown methylation status. A biopsy was performed, when possible, during the LITT procedure; however, the amount of tissue obtained was limited and was often mainly used for diagnosis (Supplementary Table S3). Biopsy during LITT revealed that six recurrent tumors had unmethylated *MGMT*, one had methylated *MGMT*, and five had an unknown status. Five tumors had EGFR amplification at biopsy during LITT, three were not amplified, and four were unknown. *TERT* and *PTEN* mutations were found in the tumors where the targeted sequence was obtained (Supplementary Table S3). Tissue was available in four patients who underwent re-resection after ICI treatment (Fig. 2). PD-L1 expression (range, 3.9%–55.7%) was detected in all tumors with variable expression of macrophages (CD163, range, 3.7%–16.5% and CD68, range, 18.7%–27.9%), CD8 T cells (range, 1.2%–14.1%), and leukocytes (CD45, range, 0.2%–12.9%) among other markers (Fig. 2; Supplementary Figs. S2 and S3).

### Correlative Studies

#### Bevacizumab Induced Changes in Circulating Plasma Analytes and Immune Cells

**FIGURE 3:**
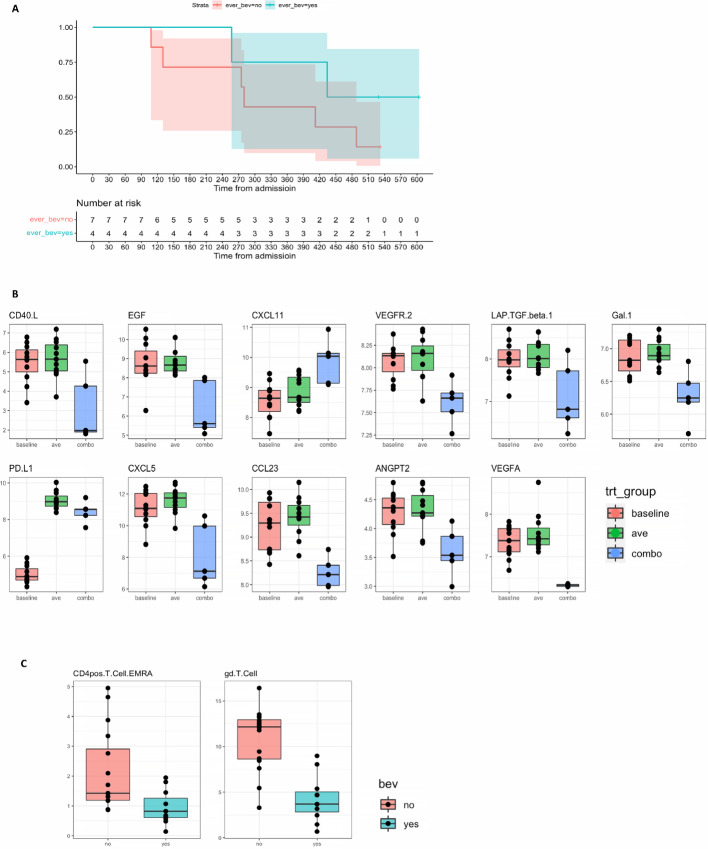
Treatment effect of combining bevacizumab with avelumab. **A,** Kaplan–Meier curves showing overall survival of patients treated with bevacizumab (blue) versus those never treated with bevacizumab (red). **B,** Difference of cytokine NPX comparing baseline, avelumab treated and avelumab combined with bevacizumab. **C,** Differentially expressed CD4^+^ T cells EMRA and γδT cells in patients’ samples treated with avelumab alone (red) combined with bevacizumab (green).

When comparing patients who received bevacizumab relative to those who did not receive bevacizumab (Fig. 3A), there was an increase in OS (HR = 0.3963, *P* = 0.116) not statistically significant, independent of sex (ANOVA, *P* = 0.5119) or race (ANOVA, *P* = 0.5197).

There were multiple significantly differentially expressed cytokines between samples from patients who were treated with avelumab and bevacizumab compared with samples from the same patients who were not treated with bevacizumab (Fig. 3B; Supplementary Table S4), despite no differences in cytokine type or levels when comparing baseline values between the two groups. In addition, MICA/MICB, which was significantly lower in patients treated with the combination of avelumab and bevacizumab relative to the same patients when only avelumab was used (*P* < 0.05), showed improved survival (HR = 0.7, *P* = 0.559) when MICA/MICB expression levels were <25% quantile. Nevertheless, there were no other differentially expressed cytokines between samples from patients who survived longer (death >400 days from enrollment) and shorter (death <400 days from enrollment).

The median PD-L1 expression in plasma samples increased by 1.8-fold after avelumab treatment and maintained the same fold difference when avelumab was combined with bevacizumab compared with baseline (*P* = 0.004).

CyTOF was used to analyze specific circulating immune cell populations. There was no difference in the circulating immune cell subsets in the samples collected at baseline or after avelumab treatment. However, there was a significant decline in γδT cells (*P* = 0.001) and CD4^+^ T cell EMRA (*P* = 0.047) between samples of patients treated with bevacizumab and samples of the same patients when they were off bevacizumab, despite no differentially expressed cell populations between baseline samples of patients treated or not treated with bevacizumab (Fig. 3C). Furthermore, when we compared the phenotypic cell frequency in patients with prolonged survival (>400 days) from enrollment in the study to those with shorter survival (<400 days), we found a significant decrease in CD4^+^ T cell EMRA (*P* = 0.007) and γδT cells (*P* = 0.042) and a significant increase in total B cells (*P* = 0.044) and specifically CD27^−^ B cells (*P* = 0.042).

## Discussion

This study showed that treating patients with the first recurrence of GBM with avelumab alone or in combination with bevacizumab and sequentially with anti-PD-1 inhibition is well tolerated, safe, and does not result in unreported adverse effects. However, the combination of LITT followed by avelumab (part B) had potential toxicity due to cerebral edema formation. The patients received dexamethasone more often and at higher doses after the LITT procedure than in part A. Baseline dexamethasone administration in patients receiving ICIs can result in decreased survival by affecting the innate and adaptive immune systems (13).

There were no radiographic responses except for 3 patients who received avelumab combined with bevacizumab. A median OS of 13 months and 14.5 months for women is encouraging, despite no improvement in PFS as suggested by other studies. Nevertheless, we must be cautious in interpreting our results as our study was prematurely closed, not allowing for the planned accrual of the expansion cohort. In addition, we may have selected patients with smaller tumors for the LITT part of the study. Several factors can negatively influence the PFS of recurrent GBM treated with ICIs: (i) pseudoprogression, as the increased size of the lesions on MRI does not reflect the underlying biological mechanisms of recruitment and infiltration of immune cells into the tumor, (ii) the low mutation burden, lack of antigen presentation, presence of suppressive cytokines, and aggressiveness of the disease does not lead to an objective response but delays tumor growth, and (iii) the heterogeneity of the tumor, including the immune components, produces an array of intermixed results that are not appreciated with current imaging modalities, but ultimately leads to improved survival. Therefore, survival may be more relevant than objective response and PFS when treating patients with GBMs with ICIs as seen in other cancers (14), and the patients may be maintained in treatment, provided they are relatively clinically stable.

Five of the 6 patients with survival >12 months received combination treatment with bevacizumab, and a PR was reached in 3. The combination of bevacizumab with ICIs decreased the levels of VEGFA and other mediators of angiogenesis such as ANGPT2 and VEGFR2. ANGPT2 participates in resistance to the targeting of VEGF-A, with high levels associated with decreased response and survival of patients on ICI treatment (15). Combining ICI with bevacizumab decreases CD68^+^ and CD163^+^ macrophages in tumors (15). Precluding the paracrine effect of VEGF signaling triggers the mobilization of CD8^+^ T cells into the TME with the destruction of tumor cells (16). In addition, our research demonstrated that bevacizumab combined with ICIs modulated inflammatory chemokines such as galectin-1 (Gal1), CXCL5, CXCL11, CCL23, CD40.L, a member of the TNF family, and TGFβ. TGFβ is a well-known potent immunosuppressive cytokine in GBM that, among other functions, promotes immune evasion by impairing the cytotoxicity activity of the natural killer innate immune system (17). Gal1 has been implicated in cancer progression by promoting angiogenesis, tumor cell proliferation, and decreasing immune cell recruitment (18). CXCL5/CXCR2 pathway facilitates the development of metastatic disease in other cancers (19). We found the stress MICA/MICB proteins to be downregulated in patients on bevacizumab, and low levels correlated with more prolonged survival, as found in other cancers (20). MICA/MICB are transmembrane glycoproteins that, in tumor evasion, undergo proteolytic shedding and function as a soluble decoy preventing activation through cell-membrane binding by quenching NKG2D, a receptor that, during cellular stress, gets activated and leads to cytotoxicity (21).

Our patients with lower γδ T cell levels had significantly higher survival rates. While γδ T cells are known to be cytotoxic to cancer cells by producing IFNγ and destroying tumor cells, there is increasing evidence that IL17-producing γδ T cells can promote tumor growth (22). IL17-producing γδ T cells release IL8, TNF, and GMCSF that recruits MDSCs and prompts worsening TME immunosuppression (22). In addition, the lack of IL17 correlated with decreased tumor angiogenesis in a sarcoma model (23). Further characterization of these subsets of γδ T cells may reveal a signature of survival, elucidate the favorable effect of their decrease by bevacizumab treatment, and may be a potential target for immune modulation in GBM.

Furthermore, we found prolonged survival in patients with lower CD4^+^ T cell EMRA. CD4^+^ T cell EMRA is a heterogeneous subset of effector memory T cells that reexpress CD45, provide protection against exposure to viral antigens (24) and appear after the acute phase of infection (25). CD4^+^ T cell EMRA and CD8^+^ T cell EMRA have been preferentially found in circulation more than in tumors. Although their role has not yet been defined in patients with cancer (26), coexpression of markers on subsets of CD4^+^ T cell EMRA thought to be due to exhaustion was associated with AML relapse (27). Our study suggests improved survival in patients with higher total B cells and CD27^−^ B cells. The role of B cells in tumor immunity is being unraveled, and their density in the TME and location in tertiary lymphoid structures might predict better prognosis and response to immunotherapy (28). The pool of CD27^−^ B cells in circulation in adults corresponds to naïve B cells (29), and levels increase in autoimmune diseases and the elderly. Moreover, CD27^−^ B cells can be precursors of CD27^+^ B cells and vice versa, and the loss or failure to upregulate CD27 in cells correlates with longer, more acidic, and hydrophobic Ab binding sites (30), and a subset of these cells have been associated with prolonged survival in lung cancer (31). Although our correlative biomarker analysis with survival is mostly hypothesis generating due to the small sample size, it may indicate a trend. Immunotherapy studies with a small sample may help identify signals of response to treatment and potential biomarkers that will require further investigation, as has been advocated (32). Our differentially expressed cytokines and immune cells findings can lead to further in-depth studies to explore, elucidate, and validate response signatures that help stratify patients and design successful combinatorial treatments.

## Supplementary Material

Table TS1 Table S1. Representativeness of Study Participants

Table TS2 Table S2. Key Antibodies Resource

Table TS3 Tumor molecular characteristics

Table TS4 One-way ANOVA analysis comparing expression of cytokines between Avelumab treated and Avelumab combined with Bevacizumab

Suppl Fig FS1 Sex as a variable of survival

Suppl Fig FS2 Chromogenic multiplex expression analysis of different biomarkers in formalin-fixed paraffin-embedded tissue of a patient's tumor when diagnosed with glioblastoma (34369), before treated with LITT (34370) and after treatment with LITT and avelumab (34371) and quantitative analysis using Halo® Image Analysis Platform.

Suppl Fig FS3 Chromogenic multiplex expression analysis of different biomarkers in formalin-fixed paraffin-embedded tissue of 34364, 34368, 34369, 34370, and 34371 tumors and quantitative analysis using Halo® Image Analysis Platform
