# Supplementary material for: A Phase I Trial of VEGF-A Inhibition Combined with PD-L1 Blockade for Recurrent Glioblastoma
Source: Cancer Res Commun. 2023 Jan 25;3(1):130–9. doi: 10.1158/2767-9764.CRC-22-0420 (PMC10035521; doi:10.1158/2767-9764.CRC-22-0420)
Supplement: Table TS1 — Table S1. Representativeness of Study Participants [file crc-22-0420-s01.docx]

**Supplementary Table S1.** Representativeness of Study Participants

| Cancer Type/Subtype | Brain/Glioblastoma-IDH wildtype |
| --- | --- |
| Epidemiology | GBM is the most frequent malignant brain tumor (approximately 50% of all primary malignant brain tumors) |
| Sex | The male-to-female ratio is 1.6:1 |
| Age | Any age, but preferential affects patients aged 55-85 years, median age 64 |
| Race/ethnicity | In the USA, incidence is higher in whites (non-Hispanic) than Hispanic whites (30% lower), blacks and Asians or Pacific Islanders (approximately 50% lower), American Indians or Alaska Natives (approximately 60% lower) |
| Geography | In USA the annual incidence is 3-4 cases per 100,000 population |
