# Supplementary material for: A Phase I Trial of VEGF-A Inhibition Combined with PD-L1 Blockade for Recurrent Glioblastoma
Source: Cancer Res Commun. 2023 Jan 25;3(1):130–9. doi: 10.1158/2767-9764.CRC-22-0420 (PMC10035521; doi:10.1158/2767-9764.CRC-22-0420)
Supplement: Table TS2 — Table S2. Key Antibodies Resource [file crc-22-0420-s02.docx]

**Supplementary** **Table S2. Key Antibodies Resource**

| **Antibody** | **Clone** | **Catalogue number** | **Source** | **Dilution** |
| --- | --- | --- | --- | --- |
| Ki-67 | 30-9 | 790-4286 | Roche Diagnostics | Prediluted |
| CD3 | 2-GV6 | 790-4341 | Roche Diagnostics | Prediluted |
| CD163 | MRQ-26 | 760-4437 | Roche Diagnostics | Prediluted |
| CD45 | 2B-11 and PD/26 | 760-4279 | Roche Diagnostics | Prediluted |
| CD68 | KP-1 | 790-2931 | Roche Diagnostics | Prediluted |
| CD8 | SP57 | 790-4460 | Roche Diagnostics | Prediluted |
| CD31 | JC70 | 760-4378 | Roche Diagnostics | Prediluted |
| PD-L1 | 28-8 | AB205921 | Abcam | 1:50 |
