# Supplementary material for: A Phase I Trial of VEGF-A Inhibition Combined with PD-L1 Blockade for Recurrent Glioblastoma
Source: Cancer Res Commun. 2023 Jan 25;3(1):130–9. doi: 10.1158/2767-9764.CRC-22-0420 (PMC10035521; doi:10.1158/2767-9764.CRC-22-0420)
Supplement: Table TS3 — Tumor molecular characteristics [file crc-22-0420-s03.pptx]

## Slide 1
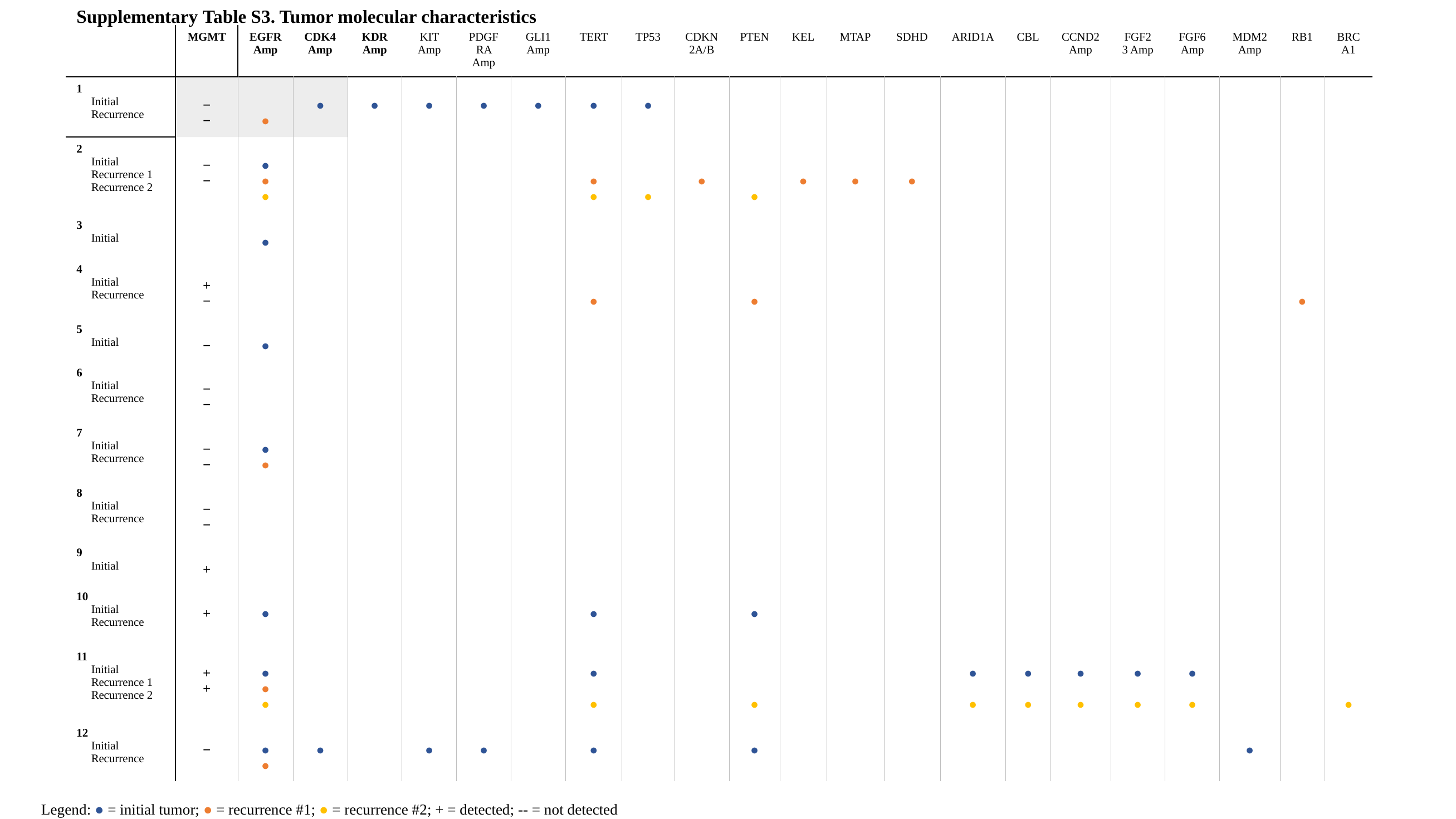

Supplementary Table S3. Tumor molecular characteristics
| | MGMT | EGFR Amp | CDK4 Amp | KDR Amp | KIT Amp | PDGFRA Amp | GLI1 Amp | TERT | TP53 | CDKN2A/B | PTEN | KEL | MTAP | SDHD | ARID1A | CBL | CCND2 Amp | FGF23 Amp | FGF6 Amp | MDM2 Amp | RB1 | BRCA1 |
| --- | --- | --- | --- | --- | --- | --- | --- | --- | --- | --- | --- | --- | --- | --- | --- | --- | --- | --- | --- | --- | --- | --- |
| 1 Initial Recurrence | − − | ● | ● | ● | ● | ● | ● | ● | ● | | | | | | | | | | | | | |
| 2 Initial Recurrence 1 Recurrence 2 | − − | ● ● ● | | | | | | ● ● | ● | ● | ● | ● | ● | ● | | | | | | | | |
| 3 Initial | | ● | | | | | | | | | | | | | | | | | | | | |
| 4 Initial Recurrence | + − | | | | | | | ● | | | ● | | | | | | | | | | ● | |
| 5 Initial | − | ● | | | | | | | | | | | | | | | | | | | | |
| 6 Initial Recurrence | − − | | | | | | | | | | | | | | | | | | | | | |
| 7 Initial Recurrence | − − | ● ● | | | | | | | | | | | | | | | | | | | | |
| 8 Initial Recurrence | − − | | | | | | | | | | | | | | | | | | | | | |
| 9 Initial | + | | | | | | | | | | | | | | | | | | | | | |
| 10 Initial Recurrence | + | ● | | | | | | ● | | | ● | | | | | | | | | | | |
| 11 Initial Recurrence 1 Recurrence 2 | + + | ● ● ● | | | | | | ● ● | | | ● | | | | ● ● | ● ● | ● ● | ● ● | ● ● | | | ● |
| 12 Initial Recurrence | − | ● ● | ● | | ● | ● | | ● | | | ● | | | | | | | | | ● | | |
Legend: ● = initial tumor; ● = recurrence #1; ● = recurrence #2; + = detected; -- = not detected
