# Supplementary material for: A Phase I Trial of VEGF-A Inhibition Combined with PD-L1 Blockade for Recurrent Glioblastoma
Source: Cancer Res Commun. 2023 Jan 25;3(1):130–9. doi: 10.1158/2767-9764.CRC-22-0420 (PMC10035521; doi:10.1158/2767-9764.CRC-22-0420)
Supplement: Table TS4 — One-way ANOVA analysis comparing expression of cytokines between Avelumab treated and Avelumab combined with Bevacizumab [file crc-22-0420-s04.pptx]

## Slide 1
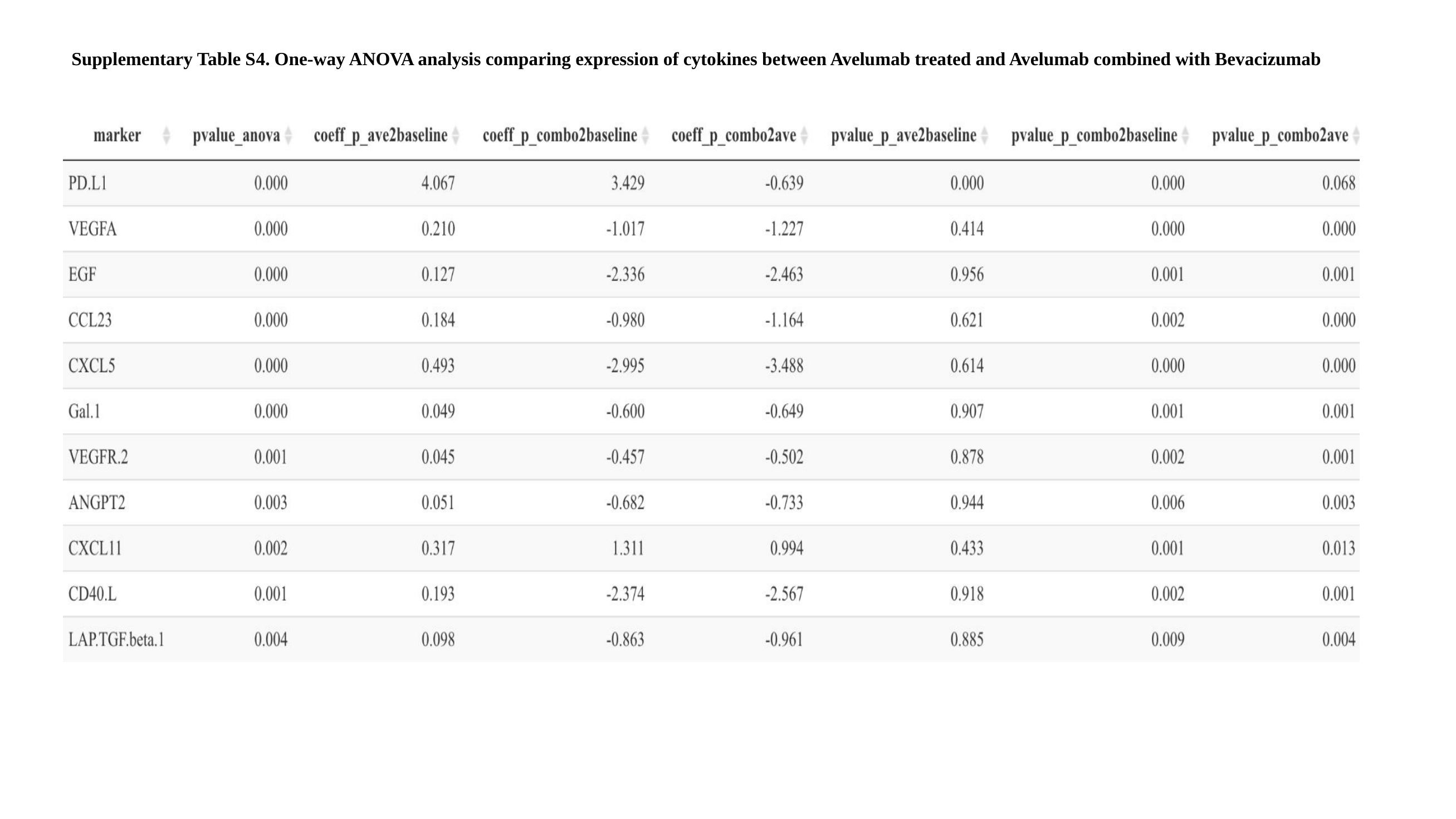

Supplementary Table S4. One-way ANOVA analysis comparing expression of cytokines between Avelumab treated and Avelumab combined with Bevacizumab
