# Supplementary material for: A Phase I Trial of VEGF-A Inhibition Combined with PD-L1 Blockade for Recurrent Glioblastoma
Source: Cancer Res Commun. 2023 Jan 25;3(1):130–9. doi: 10.1158/2767-9764.CRC-22-0420 (PMC10035521; doi:10.1158/2767-9764.CRC-22-0420)
Supplement: Suppl Fig FS1 — Sex as a variable of survival [file crc-22-0420-s05.pptx]

## Slide 1
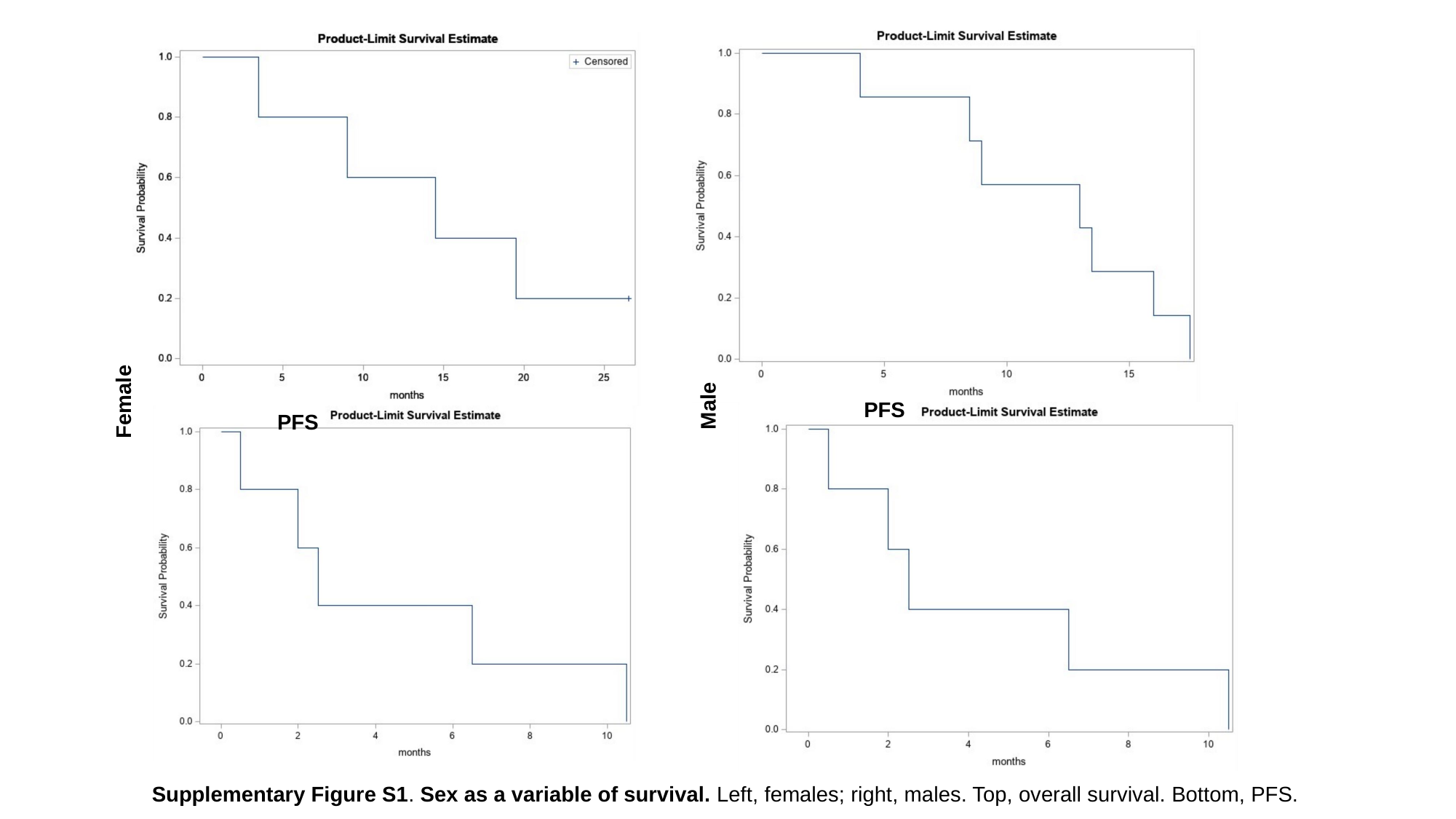

Male
Female
PFS
PFS
Supplementary Figure S1. Sex as a variable of survival. Left, females; right, males. Top, overall survival. Bottom, PFS.
