# Supplementary material for: A Phase I Trial of VEGF-A Inhibition Combined with PD-L1 Blockade for Recurrent Glioblastoma
Source: Cancer Res Commun. 2023 Jan 25;3(1):130–9. doi: 10.1158/2767-9764.CRC-22-0420 (PMC10035521; doi:10.1158/2767-9764.CRC-22-0420)
Supplement: Suppl Fig FS2 — Chromogenic multiplex expression analysis of different biomarkers in formalin-fixed paraffin-embedded tissue of a patient's tumor when diagnosed with glioblastoma (34369), before treated with LITT (34370) and after treatment with LITT and avelumab (34371) and quantitative analysis using Halo® Image Analysis Platform. [file crc-22-0420-s06.pdf]

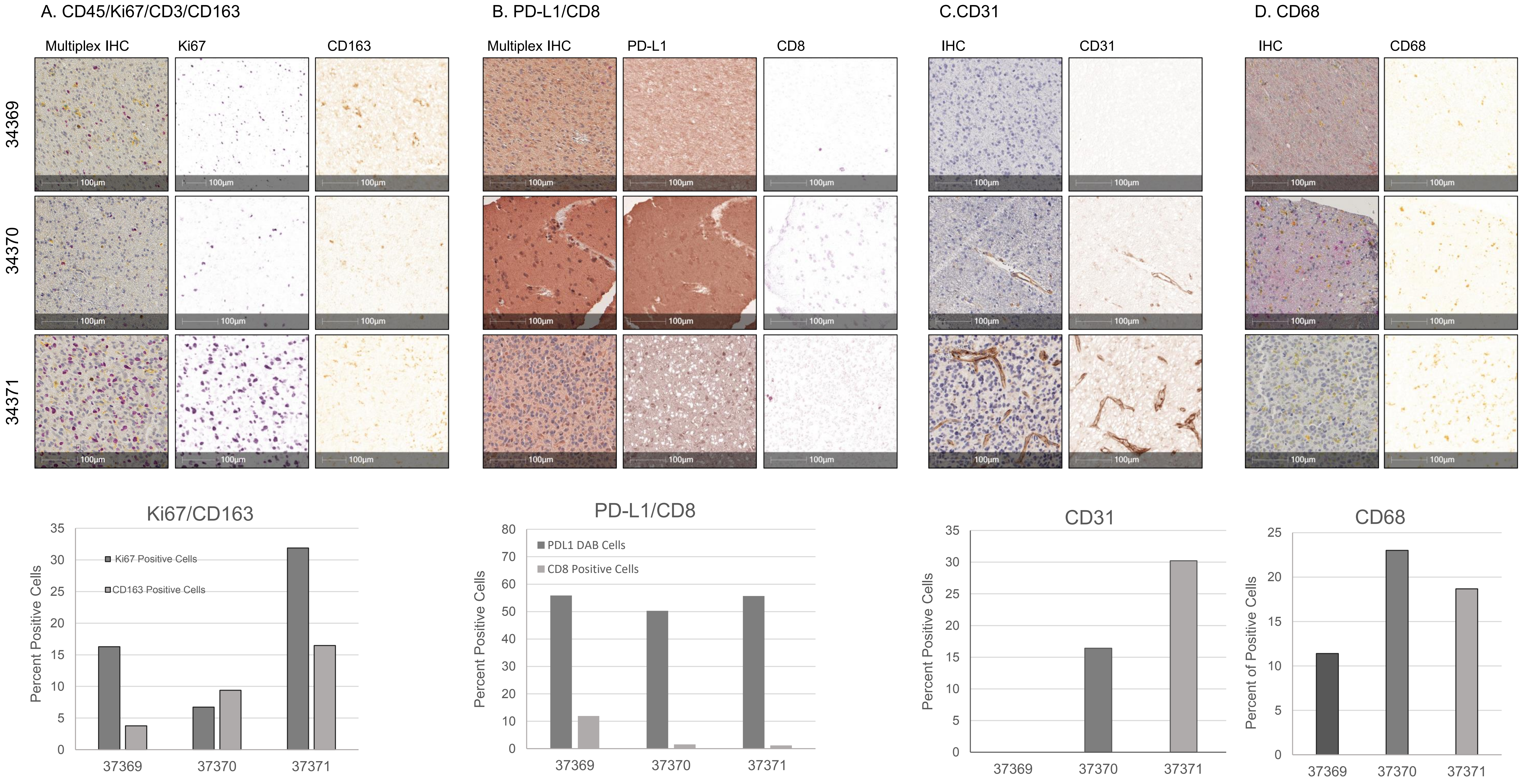

Supplementary Figure 2. Chromogenic multiplex expression analysis of different biomarkers in formalin-fixed paraffin-embedded tissue of a patient's tumor when diagnosed with glioblastoma (34369), before treated with LITT (34370) and after treatment with LITT and avelumab (34371) and quantitative analysis using Halo® Image Analysis Platform. **A**. Representative fields for chromogenic multiplex for CD45(brown)/Ki67(purple)/CD3(teal)/CD163(yellow) for 34369, 34370 and 34370. Color deconvolution for Ki67 and CD163 was obtained using HALO Image Analysis, and quantitative analysis for Ki67 and CD163 is shown below. **B.** Representative fields for chromogenic multiplex for PD-L1 (brown)/CD8 (purple) with their respective color deconvolution images and quantification are shown below. **C.** Representative fields for CD31 IHC in brown and color deconvolution images with quantitative analysis shown below and **D.** Representative fields for CD68 IHC in yellow with image color deconvolution with quantitative analysis shown below
