# Supplementary material for: A Phase I Trial of VEGF-A Inhibition Combined with PD-L1 Blockade for Recurrent Glioblastoma
Source: Cancer Res Commun. 2023 Jan 25;3(1):130–9. doi: 10.1158/2767-9764.CRC-22-0420 (PMC10035521; doi:10.1158/2767-9764.CRC-22-0420)
Supplement: Suppl Fig FS3 — Chromogenic multiplex expression analysis of different biomarkers in formalin-fixed paraffin-embedded tissue of 34364, 34368, 34369, 34370, and 34371 tumors and quantitative analysis using Halo® Image Analysis Platform [file crc-22-0420-s07.pdf]

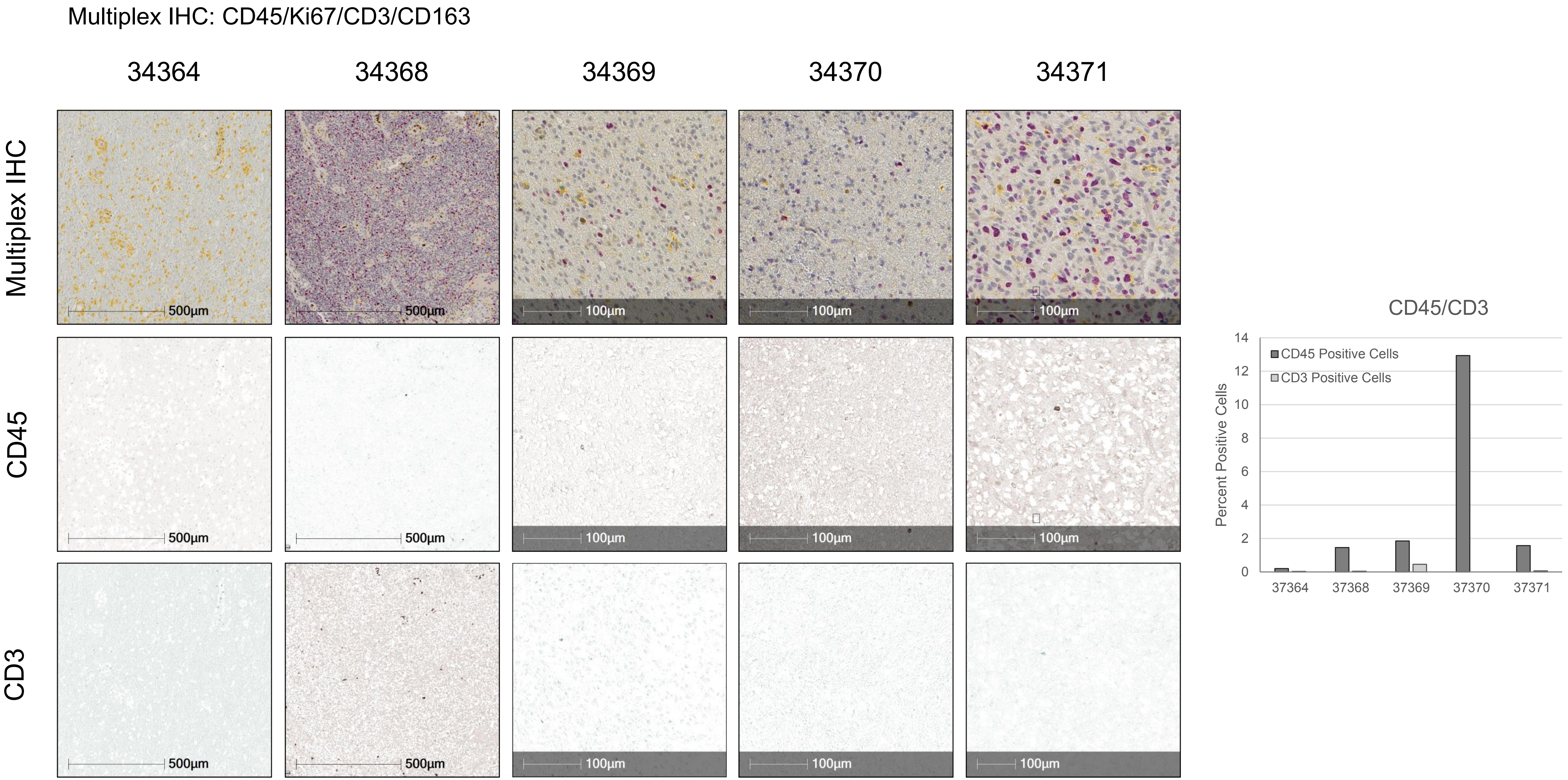

Supplementary Figure 3. Chromogenic multiplex expression analysis of different biomarkers in formalin-fixed paraffin-embedded tissue of 34364, 34368, 34369, 34370, and 34371 tumors and quantitative analysis using Halo® Image Analysis Platform. Representative fields for chromogenic multiplex for CD45(brown)/Ki67(purple)/CD3(teal)/CD163(yellow) showing the color deconvolution image for CD45 and CD3 and their quantitative analysis.
